# Noncoding RNAs in Diabetic Nephropathy: Pathogenesis, Biomarkers, and Therapy

**DOI:** 10.1155/2020/3960857

**Published:** 2020-06-19

**Authors:** Jiarong Lv, Yu Wu, Yifeng Mai, Shizhong Bu

**Affiliations:** ^1^Diabetes Research Center, Medical School of Ningbo University, Ningbo, 315000 Zhejiang, China; ^2^The Affiliated Hospital of Medical School of Ningbo University, Ningbo, 315000 Zhejiang, China

## Abstract

The correlation between diabetes and systematic well-being on human life has long established. As a common complication of diabetes, the prevalence of diabetic nephropathy (DN) has been increasing globally. DN is known to be a major cause of end-stage kidney disease (ESKD). Till now, the molecular mechanisms for DN have not been fully explored and the effective therapies are still lacking. Noncoding RNAs are a class of RNAs produced by genome transcription that cannot be translated into proteins. It has been documented that ncRNAs participate in the pathogenesis of DN by regulating inflammation, apoptosis, autophagy, cell proliferation, and other pathological processes. In this review, the pathological roles and diagnostic and therapeutic potential of three types of ncRNAs (microRNA, long noncoding RNA, and circular RNA) in the progression of DN are summarized and illustrated.

## 1. Introduction

Diabetes is a common chronic metabolic disease which has affected about half a billion people in the world. The expansion of urbanization, unhealthy diets, and sedentary lifestyles have led to an increase in the incidence of diabetes. The International Diabetes Federation (IDF) estimates that the number of diabetic patients will increase to 629 million in 2045 [1]. Diabetes is not only a health crisis but also a global social disaster. The prevalence of diabetes will become a global health and economic burden, especially in low- and middle-income countries [2].

**Figure 1:**
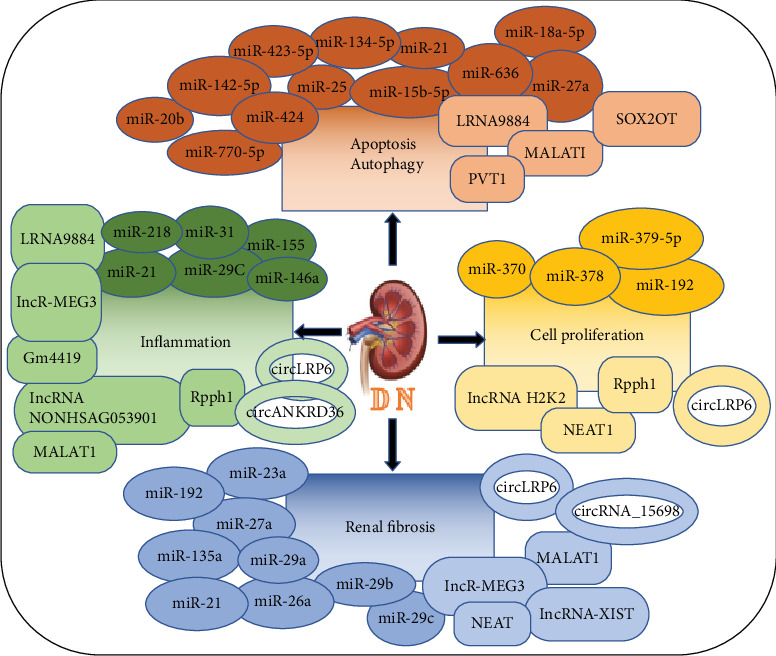
ncRNAs, including miRNAs, lncRNAs, and circRNAs, play important roles in regulating renal inflammation, apoptosis, autophagy, cell proliferation, and renal fibrosis in DN. The ellipse, the rectangle, and the ring, respectively, represent the miRNA, lncRNA, and circRNA.

Diabetic nephropathy (DN) affects approximately one-third of DM patients. As the number of patients with diabetes increases, the prevalence of patients with DN rises sharply [3]. Currently, DN is listed as the main cause of end-stage renal disease (ESKD) in the Western world [4]. The major causes of DN are glucose metabolism disorder, oxidative stress, and renal hemodynamic changes [5, 6]. However, its specific molecular mechanism remains unclear, and there is still a lack of effective therapies. Recently, there is increasing evidence that altered noncoding RNAs (ncRNAs), especially microRNAs (miRNAs), are closely related to the occurrence and progression of DN [7–10]. ncRNAs are involved in biological processes, gene expression, cell-cycle control, differentiation, and immune responses, which play important roles in various molecular biological processes in DN [11]. Many studies have found that ncRNAs play a vital role in diabetic nephropathy glomerular podocyte injury, renal tubular epithelial cell injury, glomerular mesangial cell proliferation and fibrosis, glomerular extracellular matrix accumulation, microvascular disease, endoplasmic reticulum stress and inflammation reaction, and other pathophysiological processes [12–14]. Considering the role of ncRNAs in inflammation, cell autophagy and apoptosis, and cell proliferation, some ncRNAs have also been suggested as diagnostic markers and therapeutic targets for DN [15, 16]. In this review, we summarize the pathological roles of three types of ncRNAs (microRNA, long noncoding RNA, and circular RNA) in the progression of DN (Figure 1) and illustrate their diagnostic and therapeutic potential in this disease.

## 2. Pathophysiology of DN

Kidneys are a pair of lentil-shaped organs, located in the shallow fossa on both sides of the retroperitoneal spine. The nephron is the basic unit of kidney structure and function. Each nephron includes three parts: glomerulus, renal capsule, and renal tubule [17]. The development of diabetic patients to DN is a very complicated process and is caused by a variety of factors. The major renal structural changes in DN include mesangial hyperplasia, thickening of the glomerular and tubular basement membrane, and glomerular sclerosis. The clinical manifestation of DN includes persistent proteinuria, increased blood pressure, and edema [18]. The causes of DN involve changes in renal hemodynamics, hypoxia and excessive activation of the renin-angiotensin-aldosterone system (RAAS), oxidative stress, inflammation, mitochondrial dysfunction, podocyte autophagy, and genetic and epigenetic regulation [19]. The stimulation of the RAAS system will promote the production of reactive oxygen species (ROS), thereby further damaging podocytes and renal tubular cells and aggravating kidney damage [20]. The activation of growth factors such as transforming growth factor-*β* (TGFB) and inflammatory cytokines leads to the activation of tumor necrosis factor-*α* (TNFA) signals and promotes cell repair and remodeling, thereby further aggravating kidney disease and fibrosis [19]. Animal and cell experiments have shown that ncRNA is closely related to the inflammatory response, autophagy, and gene regulation during the occurrence of diabetic nephropathy [19]. Increased understanding of diabetic nephropathy in the direction of ncRNA will help us better screen treatment strategies to prevent kidney damage associated with diabetes.

## 3. Characteristics of ncRNAs

The Encyclopedia of DNA Elements (ENCODE) consortium shows that only 1-2% of the human genome encodes proteins and another 98% consists of noncoding sequences which are biologically active and functional. An important component of the noncoding sequence is ncRNA [21]. ncRNAs are a class of RNAs produced by genome transcription but cannot be translated into proteins [22]. ncRNAs were originally thought to be a useless transcript. Fortunately, the development of high-throughput sequencing technology has led to the discovery of more ncRNAs. In addition, more and more evidence showed that ncRNAs play important roles in gene regulation processes such as DNA replication, DNA transcription, RNA translation, and RNA splicing [23, 24]. There are many types of ncRNAs, and the main classes of functional ncRNAs include microRNA (miRNA), long noncoding RNA (lncRNA), and circular RNA (circRNA), which have captured the interest of biomedical researchers and have been studied in depth [25, 26]. The discovery of noncoding RNAs (ncRNAs) opens up new prospects for DN diagnosis, prognosis, and treatment.

## 4. miRNA in DN

miRNAs are small, single-stranded RNAs, spanning about 22 nucleotides, which are generated from pre-miRNAs by RNA polymerase II. Mature miRNAs can inhibit protein expression via disruption of translation initiation or elongation by interacting with the untranslated region (UTR) of their target mRNA. miRNAs have also been shown to activate gene expression under some conditions [27]. The available evidence indicates that miRNAs are involved in the pathogenesis of DN [12, 28]. By using microarray analysis, miRNA expression profiles in human and other DN animal models have been reported [29, 30]. The latest evidence suggests that miRNA levels have changed significantly in the course of DN. For instance, DN progressors showed significantly greater levels of miR-21, miR-29a, miR-29b, and miR-29c in comparison with nonprogressors [31]. On the other hand, functional studies also indicated that knockout or upregulation of certain miRNAs was able to change the progression [32–34].

### 4.1. Role of miRNAs in Inflammation in DN

Chronic low-grade inflammation is a hallmark of type 2 diabetic nephropathy and contributes to the pathogenesis and progression of DN. A study has shown that the accumulation of inflammatory cells and proinflammatory cytokines produced by inflammatory cells in the kidney is a key player in the pathogenesis of DN [35]. A study showed that miR-29c was increased in the patients with DN, and overexpression of miR-29c in podocytes could result in an increase in inflammatory cytokines such as interleukin- (IL-) 1, IL-6, IL-18, and tumor necrosis factor- (TNF-) *α*. However, inhibition of miRNA-29c with its inhibitor reduced the inflammatory cytokines in podocytes. This indicated that miRNA-29c may contribute to the development and progression of DN as a promotive factor [12]. miR-146a is highly expressed in many cell types and plays an important anti-inflammatory role in myeloid cells. Lee et al. found that miRNA-146a produced by podocytes can inhibit inflammation by acting on their targets Notch-1 and ErbB4 [36]. The level of miR-155 is largely increased in kidney tissues of patients with DN. Furthermore, the overexpression of miRNA-155 in human renal glomerular endothelial cells will increase the expression of TNF-*α*, TGF-*β*1, and NF-*κ*B and contribute to inflammation-mediated glomerular endothelial injury [37]. miRNA-31, an inflammation-related miRNA, significantly downregulated during the progression of DN, worsens inflammation by reducing leukocyte rolling velocity and enhancing leukocyte adhesion to the endothelium, and thereby aggravates the development of the disease [38]. miRNA-21 is increased in kidney tissue, and it may be also involved in the regulation of inflammation and renal fibrosis in a DN mouse model. Overexpression of miR-21 promoted increases in the levels of proinflammatory markers and increased the amount of macrophage infiltration into diabetic kidneys, whereas knockdown of miR-21 can block the progression of renal fibrosis and inflammation, because suppression of miR-21 may inhibit the activation of the TGF-*β* and NF-*κ*B signaling pathways [33, 39]. A recent study showed that in a DN rat model, overexpressing miR-218 was sufficient to reduce renal injury through regulating NF-*κ*B-mediated inflammation. Moreover, they confirmed that miR-218 targets the messenger RNA (mRNA) encoding IKK-*β* [40].

### 4.2. Role of miRNAs in Apoptosis and Autophagy in DN

Apoptosis and autophagy are two common forms of programmed cell death and participate in the development of DN [41, 42]. There are a number of miRNAs involved in controlling programmed cell death during DN. For instance, miR-15b-5p, an apoptosis-related miRNA decreased in the patients with DN [43], can mitigate high glucose- (HG-) induced apoptosis in human kidney-2 (HK-2) cells by way of decreasing the levels of active caspase-3 and cleaved PARP. Moreover, enforced expression of miR-15b-5p can restrain the HG-stimulated inflammatory response [44]. It is reported that miR-25 can protect against high glucose-induced tubular epithelial cell damage, which is correlated with attenuated oxidative stress and apoptosis. Mechanistically, phosphatase and tensin homolog deleted on chromosome ten (PTEN) is identified as a target gene for miR-25; in this setting, overexpression of miR-25 would protect HK-2 cells against HG-induced reactive oxygen species (ROS) accumulation and apoptosis [13]. miR-21 was highly expressed in serum and kidney tissues of DN patients, and it may be also involved in the regulation of cell apoptosis and autophagy in DN [45]. Loss of miR-21 significantly repressed apoptosis and promoted autophagy, whereas overexpression of miR-21 induced cell apoptosis and curbed cell autophagy because miR-21 can inhibit forkhead box O1 (FOXO1) expression in HG-cultured podocytes [46]. In the kidney tissues of DN rats, the expression of miR-424 was significantly decreased, and the upregulation of miR-424 could significantly decrease the apoptosis rate of tissue cells by decreasing the expression levels of caspase-3 and Bax and increasing the level of the B cell lymphoma-2 gene (Bcl-2). Eventually, it improved DN symptoms. Furthermore, they found that Rictor was the direct target for miR-424, and upregulation of miR-424 inhibited Rictor through Akt signaling in renal tissue of DN rats [10]. miR-320a triggers apoptosis in podocytes by directly targeting MafB while overexpression of miR-320a aggravated renal dysfunction in db/db mice [47]. miR-770-5p is upregulated in podocytes under HG condition, and it targeted TP53-regulated inhibitor of apoptosis 1 (TRIAP1) to regulate cell apoptosis. Inhibition of miR-770-5p could promote the proliferation of podocytes and inhibited the apoptosis of podocytes [48]. A recent study identified that miR-134-5p, which binds to the 3′-untranslated region of Bcl-2, promoted high glucose-induced podocyte apoptosis in db/db mice [49]. miR-423-5p, a decreased miRNA in renal tissues of DN patients, is reported to target nicotinamide adenine dinucleotide phosphate oxidase 4 (Nox4) for inhibiting ROS production, suppressing cell apoptosis, and reducing inflammatory activity [50]. Additionally, miR-20b may also target sirtuin 7 (SIRT7), which is proven to contribute to high glucose-induced podocyte apoptosis. In this setting, miR-20b may mediate the occurrence of DN [51].

Autophagy deficiency or insufficiency in renal cells was found to contribute to the pathogenesis of diabetic nephropathy [52]. miRNA may target autophagy-related genes and signaling pathways to delay DN processes [53]. Level of miR-27a is downregulated in HK-2 cells under HG condition; this study also indicated that arbutin can protect HK-2 cells against high glucose-induced apoptosis and autophagy by upregulating microR-27a [54]. Additionally, a recent study demonstrated an increase in miR-636 expression level of renal tissues in diabetic rats during the progression of diabetes. The expression of miR-636 can be inhibited by using caffeic acid with subsequent induction of autophagy which ameliorated glomerular changes in STZ -induced diabetic rats [55]. Moreover, Xu et al. found that miR-18a-5p could regulate autophagy by targeting atactic telangiectasis mutation (ATM) [56]. miR-142-5p is upregulated in the DN model in vitro and in vivo. PTEN was found to be a downstream target of miR-142-5p. Meanwhile, downregulation of miR-142-5p could enhance autophagy, thereby inhibiting HG-induced fibrosis [57].

### 4.3. Role of miRNAs in Cell Proliferation in DN

Cell proliferation, especially the proliferation of glomerular mesangial cells (MCs), is the main pathological change of DN [58]. Some DN-associated miRNAs are involved in cell proliferation. For instance, miR-370, which is highly increased in the renal DN model, is capable of triggering cell proliferation by targeting canopy 1 (CNPY1) [28]. On the other hand, level of miR-192 is reported to be elevated in high glucose-treated rat MCs, and it regulates MC proliferation and renal fibrosis [59]. Moreover, a recent study showed that miR-379-5p has a critical role in renal fibrosis during DN. miR-379-5p was downregulated by HG treatment in mouse MCs. However, transfection with miR-379-5p mimics suppressed the proliferation and the accumulation of extracellular matrix components [60]. Nevertheless, miR-378 is highly decreased in DN and regulates the expression of fibrotic genes and the MAPK1 pathway. Overexpression of miR-378 could inhibit MC expansion and proliferation, thereby attenuating kidney cell fibrosis and mesangial hypertrophy [61]. These findings suggested that miRNAs are involved in the development process of DN through modulating cell proliferation.

### 4.4. Role of miRNAs in Renal Fibrosis of DN

At the histological level, irreversible glomerular fibrosis and scar formation are the most important pathophysiological changes during the progression of DN to end-stage renal disease. Glomerular lesions, especially fibrosis of the renal tubules and renal interstitium, also played an important role in the progress of DN. Further researches also indicate that miRNA was found to play an important role in the process of renal fibrosis during DN [62]. A recent study suggested that miR-21 can promote the development of renal fibrosis by regulating the metabolic pathways involved in fatty acid and lipid oxidation [63]. McClelland et al. found that the upregulation of miR-21 positively correlates with the severity of fibrosis and rate of decline in renal function in DN [64]. It has been confirmed that targeting of the zinc finger E-box binding homeobox 1/2 (ZEB1/2) by miR-192 has been shown to result in renal fibrosis by activation of the TGF-*β* signaling pathway [65], while Ebadi et al. suggest that enhancing the expression of miR-192 can improve DN and modulate the risk of renal function decline by suppressing fibrogenesis. Meanwhile, they found that the increase in miR-29a/b/c expression in the DN kidney tissue is able to improve DN probably via targeting the TGF-*β*/Smad signaling pathway [14]. Moreover, a recent study showed that miR-26a was downregulated in DN and it may target connective tissue growth factor (CTGF) to inhibit TGF-*β*-induced extracellular matrix protein expression in podocytes [66]. In addition, upregulation of miR-135a is detected in serum and renal tissue from patients with DN, as well from db/db mice. Inhibition of TRPC1 levels to prevent Ca^2+^ entry into cells may be a mechanism by which miR-135a promotes renal fibrosis in diabetic kidney injury [67]. Overexpression of miR-23a and miR-27a in muscle prevents diabetes-induced muscle cachexia and attenuates renal fibrosis lesions through muscle-kidney crosstalk. It may provide a new approach to the treatment of diabetic muscular atrophic nephropathy [68].

## 5. lncRNAs in DN

lncRNAs are a class of RNA transcripts, which are greater than 200 bp in length. lncRNAs are usually 5′-capped, spliced, and polyadenylated; therefore, they have major features with mRNA but usually do not contain open reading frames with translational capabilities. Compared with mRNAs, they were characterized by more space-time specificity and lower interspecies conservation [69]. lncRNAs can be grouped into sense lncRNA, antisense lncRNA, bidirectional lncRNA, intron lncRNA, intergenic lncRNA, and enhancer lncRNA by their relative location to protein-encoding genes in the genome [70]. lncRNAs exert diverse biological functions by controlling nuclear architecture and transcription in the nucleus and modulating mRNA stability, translation, and posttranslational modifications in the cytoplasm [69].

lncRNAs are newly identified intracellular noncoding ribonucleotides that regulate different biological activities in different organs including the kidney [71]. Yang et al. found 45 upregulated lncRNAs and 813 downregulated lncRNAs in the DN group compared with DM patients without microalbuminuria and healthy controls. Meanwhile, they found levels of lncRNA-ARAP1-AS2 gradually increasing during the progression of diabetes and DN whereas those of lncRNA-ARAP1-AS1 gradually decreased [15]. Till now, the biological role of lncRNAs in DN attracts more attentions. For instance, lncRNA NONHSAG053901 can directly interact with early growth response protein 1 (Egr-1) to regulate the TGF-*β* inflammasome signaling pathway and mediate inflammatory molecular expressions in MCs, and it is associated with the development of inflammation, fibrosis, and proliferation of MCs in DN [72]. Gm4419 is a newly identified proinflammatory lncRNA in kidney tissues. It has been shown that NF-*κ*B/NLRP3 inflammasome-mediated inflammation in DN can be attenuated by specific knockdown of LincR-Gm4419 [73]. Furthermore, a study indicated that lncRNA MEG3, upregulated in DN, can promote fibrosis and inflammatory response via the Egr-1/TLR4 axis in vitro and in vivo. Meanwhile, MEG3 is identified as an endogenous sponge for miR-181a by targeting in an Ago2-dependent manner [74]. LRNA9884, a newly discovered Smad3-dependent lncRNA, is highly expressed in db/db mice associated with T2DN development. It may promote diabetic kidney injury by enhancing MCP-1-dependent inflammation, and it may be a novel therapeutic target for T2DN in the future [75].

Additionally, lncRNAs are also involved in the regulations of cell death and cell proliferation. In DN, an upregulated lncRNA, LINC00462, was found to participate in high glucose-induced apoptosis of renal tubular epithelial cells by the AKT pathway. In addition, knockdown of LINC00462 inhibited HG-induced cell apoptosis and affected the expression of apoptosis-related proteins by activating the AKT pathway [76]. MALAT1 is another important lncRNA involved in DN. MALAT1 can be induced in the streptozocin-induced DN mouse model, and it plays a role in high glucose-associated podocyte damage involving a feedback loop with beta-catenin [77]. Furthermore, the upregulation of MALAT1 upon glucose exposure led to increased IL-1 and TNF-*α* levels indicating that this lncRNA may be involved in inflammatory processes during DN [78]. Moreover, Li et al. found that MALAT1 in renal tubular epithelial cells induced by high glucose leads to increased pyroptosis by targeting miR-23c and consecutive upregulation of ELAVL1 and NLRP3 [79]. Additionally, PVT1, a well-studied lncRNA, was highly expressed in primary podocytes in DN patients. The silencing of lncRNA PVT1 exerted inhibitory effects on podocyte damage and apoptosis via upregulating FOXA1 [80]. More importantly, lncRNA SOX2OT was confirmed to mitigate podocyte injury induced by the high glucose through autophagy induction by the miR-9/SIRT1 axis. Thus, SOX2OT may serve as a potential therapeutic target for DN [81]. It has been reported that lncRNA H2k2 could promote MC proliferation in DN via the miR-449a/b/Trim11/Mek signaling pathway [82]. A recent study found that NEAT1 was induced in murine MCs treated with high glucose. Overexpression of NEAT1 led to the activation of the AKT/mTOR signal path, thereby increasing cellular proliferation and fibrosis [83]. In addition, lncRNA Rpph1 may promote inflammatory response and proliferation of MCs in DN by interacting with Gal-3 and activating the Mek/Erk pathway [84].

In renal tissues of DN patients, DN mice, and high glucose-exposed HK-2 cells, lncRNA X-inactive specific transcript (XIST) was found to be highly increasing. Downregulated expression of XIST led to an increase in miR-93-5p expression, thereby decreasing CDKN1A and suppressing renal interstitial fibrosis in DN [85]. Overall, although the potential role of lncRNAs in the pathogenesis of DN has not been fully understood, emerging evidence shows the importance of lncRNAs in the progression of DN.

## 6. circRNAs and DN

circRNAs are a class of newly identified ncRNAs without either polyadenylated tails in 3′ ends or the cap structure at 5′ ends. So their structure comprises covalently closed loops, and they can be protected against RNA exonucleases [86]. Besides, accumulating investigations have found that circRNAs have many miRNA-binding sites which can help them to act as a miRNA sponge. The combination of circRNA and miRNA will lead to a reduction in the inhibitory effect of miRNAs on target genes, thereby enhancing the expression of these genes [87, 88].

At present, the research of circRNAs involved in diseases is getting more and more attention. Articles on circular RNA have grown exponentially, especially in cancer. Previous studies showed that circRNA, a large class of noncoding RNAs, functions by binding with miRNAs and terminating the regulation of their target genes [89]. The role of circular RNA in cancer has been recently reported, but with the in-depth study of circular RNA, some scholars have found that it also plays an important role in the development of DN. A recent study showed that circLRP6, a newly found ncRNA, was significantly expressed in MCs treated with HG. It was found to be a sponge for miRNA-205. HMGB1 is a potential target of miR-205. So circLRP6 can regulate HG-induced MC proliferation, oxidative stress, ECM accumulation, and inflammation by sponging miR-205, upregulating HMGB1, and activating the TLR4/NF-*κ*B pathway [90]. In addition, Hu et al. found that circRNA_15698 significantly upregulated in diabetic mice when performing circRNA microarray analysis in DN db/db mice. In the further experiments, they also found that circRNA_15698 can act as a sponge for miRNA, positively regulating the expression of the transforming growth factor-*β*1 (TGF-*β*1), thereby promoting the synthesis of extracellular matrix- (ECM-) related proteins. However, knockdown of circRNA_15698 suppressed the exposure with normal ECM accumulation of MCs [91]. By RNA sequencing of peripheral blood, Fang et al. identified that the expression of circANKRD36 was upregulated in peripheral blood leucocytes and was correlated with chronic inflammation in T2DM. It implied that circANKRD36 may be involved in the process of DN [92]. All this evidence indicates that revealing the mystery of circRNA is critical in the war against DN.

## 7. ncRNAs as Biomarkers in DN

**Figure 2:**
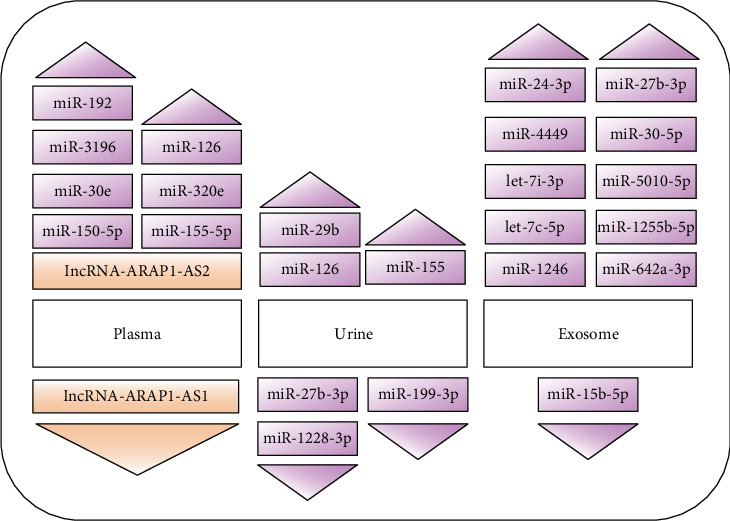
ncRNAs in plasma, urine, and exosome as a new biomarker in DN. Purple represents miRNAs, and orange represents lncRNAs.

Early detection of DN is very useful for preventing progression to renal failure. Several biomarkers of DN progression have been reported, such as peptides, growth factors, and cytokines [93]. However, due to the high stability of ncRNAs in body fluids (urine, plasma, and exosomes) and the development of detection techniques [94], they have been recognized as a new sensitive, noninvasive diagnostic biomarker for DN. Here, we summarize several biomarkers for DN (Figure 2). In plasma, miR-126 and miR-192 are downregulated in DN patients when compared with healthy controls [95]. In addition, miR-150-5p, miR-155-5p, miR-30e, miR-320e, and miR-3196 were detected to be increased in DN, and miR-150-5p and miR-155-5p were negatively correlated with the albuminuria excretion rate and positively correlated with the estimated glomerular filtration rate [16]. Kim et al. found that miR-1246, miR-642a-3p, let-7c-5p, miR-1255b-5p, let-7i-3p, miR-5010-5p, miR-150-3p, and miR-4449 were upregulated in the exosome of DN and they were all significantly correlated with the degree of albuminuria [96]. Levels of let-7i-3p, miR-24-3p, miR-30-5p, and miR-27b-3p were increasing, and the level of miR-15b-5p was decreasing in urinary extracellular vesicles of DN [97]. miRNA-27b-3p and miRNA-1228-3p were identified to be downregulated in urine, and they were found to be correlated with the progression of kidney fibrosis in DN [98]. Additionally, Meng et al. showed that decreased urinary miR-199-3p could screen DN patients from DM patients and healthy controls [99]. However, Beltrami et al. found that miR-126, miR-155, and miR-29b were upregulated in urine of DN [100]. A recent study showed that lncRNA-ARAP1-AS2 gradually increased and lncRNA-ARAP1-AS1 gradually decreased in DN/diabetes mellitus patients without microalbuminuria (DM)/healthy controls (N). They may serve as new biomarkers for diabetes and DN [15]. Due to its special ring structure, circRNA is not easily degraded in the body, and more and more scholars believe that it will be a new marker for the diagnosis of DN.

## 8. Therapeutic Potential of ncRNA in DN

Over the past few decades, ncRNAs have gained attention for their function involved in regulating signaling pathways of DN. Considering the potential involvement of different ncRNAs in the molecular signaling pathways in DN, ncRNAs are considered to be a new therapeutic target in DN. Further researches have focused on DN-specific miRNAs such as miR-21, miR-29a, miR-192, miR-188-5p, miR-200a, and miR-133b, some of which have shown encouraging therapeutic outcomes in the animal models of DN [14, 33, 101–104]. For instance, reducing miR-200a can ameliorate oxidative stress in experimental DN, and silencing of miR-21 can decrease mesangial expansion, interstitial fibrosis, macrophage infiltration, podocyte loss, albuminuria, and fibrotic and inflammatory gene expression [33, 103]. In addition, specific reduction of renal miR-27a decreases renal fibrosis, and targeting miR-27a may represent a novel therapeutic approach for DN [105]. Besides, Wu et al. found that downregulation of MALAT1 could improve renal function after duodenal-jejunal bypass in a diabetic rat model [106]. A recent research showed that silencing of lncRNA-GAS5 alleviated the HG-caused HK-2 cell toxicity [9]. Excitingly, some recent studies identified that miRNA-rich exosomes secreted by mesenchymal stem cells (MSCs) can inhibit proinflammatory cytokine expression and fibrosis in the tubulointerstitial region. At the same time, it also can inhibit epithelial-to-mesenchymal transition of renal tubular epithelial cells. Therefore, MSC therapy is considered to be a promising treatment [107]. In short, the therapeutic potential of ncRNA has been discovered, but the current findings are only the tip of the iceberg, and further research is also needed.

## 9. Conclusion

In this review, the recent progress in the involvement of ncRNAs in the pathogenesis of DN was summarized. ncRNA promotes the occurrence and development of DN through inflammation, cell apoptosis and autophagy, cell proliferation, and other pathways. miRNA-targeted therapies (including MSC and drug-targeted miRNAs) have showed encouraging outcomes; however, due to the low specificity of miRNAs to target genes, off-targeting often occurs when using miRNA-targeted therapy. Therefore, in-depth research found that a highly specific lncRNA and circRNA are imminent. It is expected that a highly specific and sensitive ncRNA may be discovered in the future and could be subsequently developed for clinical applications.

## References

[1] Sadikot S., Cho Nam Han (2017). *IDF Diabetes Atlas Eighth Edition 2017*.

[2] Cannon A., Handelsman Y., Heile M., Shannon M. (2018). Burden of illness in type 2 diabetes mellitus. *Journal of Managed Care & Specialty Pharmacy*.

[3] Reutens A. T., Atkins R. C. (2011). Epidemiology of diabetic nephropathy. *Contributions to Nephrology*.

[4] Gembillo G., Cernaro V., Salvo A. (2019). Role of vitamin D status in diabetic patients with renal disease. *Medicina*.

[5] Ni W. J., Tang L. Q., Wei W. (2015). Research progress in signalling pathway in diabetic nephropathy. *Diabetes/Metabolism Research and Reviews*.

[6] Tanaka S., Sugiura Y., Saito H. (2018). Sodium-glucose cotransporter 2 inhibition normalizes glucose metabolism and suppresses oxidative stress in the kidneys of diabetic mice. *Kidney International*.

[7] Hagiwara S., McClelland A., Kantharidis P. (2013). MicroRNA in diabetic nephropathy: renin angiotensin, aGE/RAGE, and oxidative stress pathway. *Journal Diabetes Research*.

[8] Alvarez M. L., Distefano J. K. (2013). The role of non-coding RNAs in diabetic nephropathy: potential applications as biomarkers for disease development and progression. *Diabetes Research and Clinical Practice*.

[9] Lv L., Li D., Tian F., Li X., Jing Zhang, Yu X. (2019). Silence of lncRNA GAS5 alleviates high glucose toxicity to human renal tubular epithelial HK-2 cells through regulation of miR-27a. *Artificial Cells, Nanomedicine, and Biotechnology*.

[10] Wang G., Yan Y., Xu N., Hui Y., Yin D. (2018). Upregulation of microRNA-424 relieved diabetic nephropathy by targeting Rictor through mTOR complex2/protein kinase B signaling. *Journal of Cellular Physiology*.

[11] DiStefano J. K. (2018). The emerging role of long noncoding RNAs in human disease. *Methods in Molecular Biology*.

[12] Guo J., Li J., Zhao J. (2017). MiRNA-29c regulates the expression of inflammatory cytokines in diabetic nephropathy by targeting tristetraprolin. *Scientific Reports*.

[13] Li H., Zhu X., Zhang J., Shi J. (2017). MicroRNA-25 inhibits high glucose-induced apoptosis in renal tubular epithelial cells via PTEN/AKT pathway. *Biomedicine & Pharmacotherapy*.

[14] Ebadi Z., Moradi N., Kazemi Fard T. (2019). Captopril and spironolactone can attenuate diabetic nephropathy in Wistar rats by targeting microRNA-192 and microRNA-29a/b/c. *DNA and Cell Biology*.

[15] Yang Y., Lv X., Fan Q. (2019). Analysis of circulating lncRNA expression profiles in patients with diabetes mellitus and diabetic nephropathy: differential expression profile of circulating lncRNA. *Clinical Nephrology*.

[16] Wang J., Wang G., Liang Y., Zhou X. (2019). Expression profiling and clinical significance of plasma microRNAs in diabetic nephropathy. *Journal Diabetes Research*.

[17] Schedl A. (2007). Renal abnormalities and their developmental origin. *Nature Reviews Genetics*.

[18] Sun H. J., Wu Z. Y., Cao L. (2019). Hydrogen sulfide: recent progression and perspectives for the treatment of diabetic nephropathy. *Molecules*.

[19] Lin Y. C., Chang Y. H., Yang S. Y., Wu K. D., Chu T. S. (2018). Update of pathophysiology and management of diabetic kidney disease. *Journal of the Formosan Medical Association*.

[20] Chawla T. S., Sharma D., Singh A. (2010). Role of the renin angiotensin system in diabetic nephropathy. *World Journal of Diabetes*.

[21] The ENCODE Project Consortium (2012). An integrated encyclopedia of DNA elements in the human genome. *Nature*.

[22] Cech T. R., Steitz J. A. (2014). The Noncoding RNA Revolution–Trashing Old Rules to Forge New Ones. *Cell*.

[23] Wang J., Samuels D. C., Zhao S. L., Xiang Y., Zhao Y. Y., Guo Y. (2017). Current research on non-coding ribonucleic acid (RNA). *Genes*.

[24] Fu X. D. (2014). Non-coding RNA: a new frontier in regulatory biology. *National Science Review*.

[25] Esteller M. (2011). Non-coding RNAs in human disease. *Nature Reviews Genetics*.

[26] Smith M. A., Mattick J. S. (2017). Structural and functional annotation of long noncoding RNAs. *Methods in Molecular Biology*.

[27] Bartel D. P. (2018). Metazoan microRNAs. *Cell*.

[28] Yu F. N., Hu M. L., Wang X. F. (2019). Effects of microRNA-370 on mesangial cell proliferation and extracellular matrix accumulation by binding to canopy 1 in a rat model of diabetic nephropathy. *Journal of Cellular Physiology*.

[29] Argyropoulos C., Wang K., McClarty S. (2013). Urinary microRNA profiling in the nephropathy of type 1 diabetes. *PLoS One*.

[30] Rubin A., Salzberg A. C., Imamura Y., Grivitishvilli A., Tombran-Tink J. (2016). Identification of novel targets of diabetic nephropathy and PEDF peptide treatment using RNA-seq. *BMC Genomics*.

[31] Chien H. Y., Chen C. Y., Chiu Y. H., Lin Y. C., Li W. C. (2016). Differential microRNA profiles predict diabetic nephropathy progression in Taiwan. *International Journal of Medical Sciences*.

[32] Bhatt K., Lanting L. L., Jia Y. (2016). Anti-inflammatory role of microRNA-146a in the pathogenesis of diabetic nephropathy. *J Am Soc Nephrol*.

[33] Kölling M., Kaucsar T., Schauerte C. (2017). Therapeutic miR-21 silencing ameliorates diabetic kidney disease in mice. *Molecular Therapy*.

[34] Zhu X., Zhang C., Fan Q. (2016). Inhibiting microRNA-503 and microRNA-181d with losartan ameliorates diabetic nephropathy in KKAy mice. *Medical Science Monitor*.

[35] Matoba K., Takeda Y., Nagai Y., Kawanami D., Utsunomiya K., Nishimura R. (2019). Unraveling the role of inflammation in the pathogenesis of diabetic kidney disease. *Int J Mol Sci*.

[36] Lee H. W., Khan S. Q., Khaliqdina S. (2017). Absence of miR-146a in podocytes increases risk of diabetic glomerulopathy via up-regulation of ErbB4 and Notch-1. *The Journal of Biological Chemistry*.

[37] Huang Y., Liu Y., Li L. (2014). Involvement of inflammation-related miR-155 and miR-146a in diabetic nephropathy: implications for glomerular endothelial injury. *BMC Nephrology*.

[38] Rovira-Llopis S., Escribano-Lopez I., Diaz-Morales N. (2018). Downregulation of miR-31 in diabetic nephropathy and its relationship with inflammation. *Cellular Physiology and Biochemistry*.

[39] Zhong X., Chung A. C. K., Chen H. Y. (2013). miR-21 is a key therapeutic target for renal injury in a mouse model of type 2 diabetes. *Diabetologia*.

[40] Li M., Guo Q., Cai H., Wang H., Ma Z., Zhang X. (2019). miR‐218 regulates diabetic nephropathy via targeting IKK‐*β* and modulating NK‐*κ*B‐mediated inflammation. *Journal of Cellular Physiology*.

[41] Turkmen K. (2017). Inflammation, oxidative stress, apoptosis, and autophagy in diabetes mellitus and diabetic kidney disease: the Four Horsemen of the Apocalypse. *International Urology and Nephrology*.

[42] Sifuentes-Franco S., Padilla-Tejeda D. E., Carrillo-Ibarra S., Miranda-Díaz A. G. (2018). Oxidative stress, apoptosis, and mitochondrial function in diabetic nephropathy. *International Journal of Endocrinology*.

[43] Li W., Yang S., Qiao R., Zhang J. (2018). Potential value of urinary exosome-derived let-7c-5p in the diagnosis and progression of type II diabetic nephropathy. *Clinical Laboratory*.

[44] Shen H., Fang K., Guo H., Wang G. (2019). High glucose-induced apoptosis in human kidney cells was alleviated by miR-15b-5p mimics. *Biological & Pharmaceutical Bulletin*.

[45] Chen X., Zhao L., Xing Y., Lin B. (2018). Down-regulation of microRNA-21 reduces inflammation and podocyte apoptosis in diabetic nephropathy by relieving the repression of TIMP3 expression. *Biomedicine & Pharmacotherapy*.

[46] Wang J., Shen L., Hong H., Li J., Wang H., Li X. (2019). Atrasentan alleviates high glucose-induced podocyte injury by the microRNA-21/forkhead box O1 axis. *European Journal of Pharmacology*.

[47] He M., Wang J., Yin Z. (2019). MiR-320a induces diabetic nephropathy via inhibiting MafB. *Aging (Albany NY)*.

[48] Zhang S.-Z., Qiu X. J., Dong S. S. (2019). MicroRNA-770-5p is involved in the development of diabetic nephropathy through regulating podocyte apoptosis by targeting TP53 regulated inhibitor of apoptosis 1. *European Review for Medical and Pharmacological Sciences*.

[49] Qian X., Tan J., Liu L. (2018). MicroRNA-134-5p promotes high glucose-induced podocyte apoptosis by targeting bcl-2. *American Journal of Translational Research*.

[50] Xu Y., Zhang J., Fan L., He X. (2018). miR-423-5p suppresses high-glucose-induced podocyte injury by targeting Nox4. *Biochemical and Biophysical Research Communications*.

[51] Wang X., Lin B., Nie L., Li P. (2017). MicroRNA-20b contributes to high glucose-induced podocyte apoptosis by targeting SIRT7. *Molecular Medicine Reports*.

[52] Kitada M., Ogura Y., Monno I., Koya D. (2017). Regulating autophagy as a therapeutic target for diabetic nephropathy. *Current Diabetes Reports*.

[53] Ma J., Wang Y., Xu H. T. (2019). MicroRNA: a novel biomarker and therapeutic target to combat autophagy in diabetic nephropathy. *European Review for Medical and Pharmacological Sciences*.

[54] Lv L., Zhang J., Tian F., Li X., Li D., Yu X. (2019). Arbutin protects HK-2 cells against high glucose-induced apoptosis and autophagy by up-regulating microRNA-27a. *Artificial Cells, Nanomedicine, and Biotechnology*.

[55] Salem A. M., Ragheb A. S., Hegazy M. G. A., Matboli M., Eissa S. (2019). Caffeic acid modulates miR-636 expression in diabetic nephropathy rats. *Indian Journal of Clinical Biochemistry*.

[56] Xu X. H., Ding D. F., Yong H. J. (2017). Resveratrol transcriptionally regulates miRNA-18a-5p expression ameliorating diabetic nephropathy via increasing autophagy. *European Review for Medical and Pharmacological Sciences*.

[57] Chen J., Cui Y., Zhang N., Yao X., Wang Z., Yang L. (2019). Oleanolic acid attenuated diabetic mesangial cell injury by activation of autophagy via miRNA-142-5p/PTEN signaling. *Cytotechnology*.

[58] Lei D., Chengcheng L., Xuan Q. (2019). Quercetin inhibited mesangial cell proliferation of early diabetic nephropathy through the Hippo pathway. *Pharmacological Research*.

[59] Mao Q., Chen C., Liang H., Zhong S., Cheng X., Li L. (2019). Astragaloside IV inhibits excessive mesangial cell proliferation and renal fibrosis caused by diabetic nephropathy via modulation of the TGF-*β*1/Smad/miR-192 signaling pathway. *Experimental and Therapeutic Medicine*.

[60] Li N., Wang L.‑. J., Xu W.‑. L., Liu S., Yu J.‑. Y. (2019). MicroRNA‑379‑5p suppresses renal fibrosis by regulating the LIN28/let‑7 axis in diabetic nephropathy. *International Journal of Molecular Medicine*.

[61] Wang B., Yao K., Wise A. F. (2017). miR-378 reduces mesangial hypertrophy and kidney tubular fibrosis via MAPK signalling. *Clinical Science (London, England)*.

[62] Lv W., Fan F., Wang Y. (2018). Therapeutic potential of microRNAs for the treatment of renal fibrosis and CKD. *Physiological Genomics*.

[63] Chau B. N., Xin C., Hartner J. (2012). MicroRNA-21 promotes fibrosis of the kidney by silencing metabolic pathways. *Science Translational Medicine*.

[64] McClelland A. D., Herman-Edelstein M., Komers R. (2015). miR-21 promotes renal fibrosis in diabetic nephropathy by targeting PTEN and SMAD7. *Clinical Science (London, England)*.

[65] Kato M., Arce L., Wang M., Putta S., Lanting L., Natarajan R. (2011). A microRNA circuit mediates transforming growth factor-*β*1 autoregulation in renal glomerular mesangial cells. *Kidney International*.

[66] Koga K., Yokoi H., Mori K. (2015). MicroRNA-26a inhibits TGF-*β*-induced extracellular matrix protein expression in podocytes by targeting CTGF and is downregulated in diabetic nephropathy. *Diabetologia*.

[67] He F., Peng F., Xia X. (2014). MiR-135a promotes renal fibrosis in diabetic nephropathy by regulating TRPC1. *Diabetologia*.

[68] Zhang A., Li M., Wang B., Klein J. D., Price S. R., Wang X. H. (2018). miRNA-23a/27a attenuates muscle atrophy and renal fibrosis through muscle-kidney crosstalk. *Journal of Cachexia, Sarcopenia and Muscle*.

[69] Yao R. W., Wang Y., Chen L. L. (2019). Cellular functions of long noncoding RNAs. *Nature Cell Biology*.

[70] Dahariya S., Paddibhatla I., Kumar S., Raghuwanshi S., Pallepati A., Gutti R. K. (2019). Long non-coding RNA: classification, biogenesis and functions in blood cells. *Molecular Immunology*.

[71] Mercer T. R., Mattick J. S. (2013). Structure and function of long noncoding RNAs in epigenetic regulation. *Nature Structural & Molecular Biology*.

[72] Peng W., Huang S., Shen L., Tang Y., Li H., Shi Y. (2019). Long noncoding RNA NONHSAG053901 promotes diabetic nephropathy via stimulating Egr‐1/TGF‐*β*‐mediated renal inflammation. *Journal of Cellular Physiology*.

[73] Yi H., Peng R., Zhang L. Y. (2017). LincRNA-Gm4419 knockdown ameliorates NF-*κ* B/NLRP3 inflammasome-mediated inflammation in diabetic nephropathy. *Cell Death & Disease*.

[74] Zha F., Qu X., Tang B. (2019). Long non-coding RNA MEG3 promotes fibrosis and inflammatory response in diabetic nephropathy via miR-181a/Egr-1/TLR4 axis. *Aging (Albany NY)*.

[75] Zhang Y. Y., Tang P. M. K., Tang P. C. T. (2019). LRNA9884, a novel Smad3-dependent long noncoding RNA, promotes diabetic kidney injury indb/dbMice via enhancing MCP-1-dependent renal inflammation. *Diabetes*.

[76] Wang R., Yan Y., Li C. (2019). LINC00462 is involved in high glucose‐induced apoptosis of renal tubular epithelial cells via AKT pathway. *Cell Biology International*.

[77] Hu M., Wang R., Li X. (2017). LncRNA MALAT1 is dysregulated in diabetic nephropathy and involved in high glucose-induced podocyte injuryviaits interplay with *β*-catenin. *Journal of Cellular and Molecular Medicine*.

[78] Puthanveetil P., Chen S., Feng B., Gautam A., Chakrabarti S. (2015). Long non-coding RNA MALAT1 regulates hyperglycaemia induced inflammatory process in the endothelial cells. *Journal of Cellular and Molecular Medicine*.

[79] Li X., Zeng L., Cao C. (2017). Long noncoding RNA MALAT1 regulates renal tubular epithelial pyroptosis by modulated miR-23c targeting of ELAVL1 in diabetic nephropathy. *Experimental Cell Research*.

[80] Liu D. W., Zhang J. H., Liu F. X. (2019). Silencing of long noncoding RNA PVT1 inhibits podocyte damage and apoptosis in diabetic nephropathy by upregulating FOXA1. *Experimental & Molecular Medicine*.

[81] Zhang Y., Chang B., Zhang J., Wu X. (2019). LncRNA SOX2OT alleviates the high glucose-induced podocytes injury through autophagy induction by the miR-9/SIRT1 axis. *Experimental and Molecular Pathology*.

[82] Chen W., Peng R., Sun Y. (2019). The topological key lncRNA H2k2 from the ceRNA network promotes mesangial cell proliferation in diabetic nephropathyviathe miR-449a/b/Trim11/Mek signaling pathway. *The FASEB Journal*.

[83] Huang S., Xu Y., Ge X. (2018). Long noncoding RNA NEAT1 accelerates the proliferation and fibrosis in diabetic nephropathy through activating Akt/mTOR signaling pathway. *Journal of Cellular Physiology*.

[84] Zhang P., Sun Y., Peng R. (2019). Long non-coding RNA Rpph1 promotes inflammation and proliferation of mesangial cells in diabetic nephropathy via an interaction with Gal-3. *Cell Death & Disease*.

[85] Yang J., Shen Y., Yang X. (2019). Silencing of long noncoding RNA XIST protects against renal interstitial fibrosis in diabetic nephropathy via microRNA-93-5p-mediated inhibition of CDKN1A. *American Journal of Physiology. Renal Physiology*.

[86] Jeck W. R., Sharpless N. E. (2014). Detecting and characterizing circular RNAs. *Nature Biotechnology*.

[87] Memczak S., Jens M., Elefsinioti A. (2013). Circular RNAs are a large class of animal RNAs with regulatory potency. *Nature*.

[88] Hansen T. B., Jensen T. I., Clausen B. H. (2013). Natural RNA circles function as efficient microRNA sponges. *Nature*.

[89] Kulcheski F. R., Christoff A. P., Margis R. (2016). Circular RNAs are miRNA sponges and can be used as a new class of biomarker. *Journal of Biotechnology*.

[90] Chen B., Li Y., Liu Y., Xu Z. (2019). circLRP6 regulates high glucose-induced proliferation, oxidative stress, ECM accumulation, and inflammation in mesangial cells. *Journal of Cellular Physiology*.

[91] Hu W., Han Q., Zhao L., Wang L. (2019). Circular RNA circRNA_15698 aggravates the extracellular matrix of diabetic nephropathy mesangial cells via miR-185/TGF-*β*1. *Journal of Cellular Physiology*.

[92] Fang Y., Wang X., Li W. (2018). Screening of circular RNAs and validation of circANKRD36 associated with inflammation in patients with type 2 diabetes mellitus. *International Journal of Molecular Medicine*.

[93] Fassett R. G., Venuthurupalli S. K., Gobe G. C., Coombes J. S., Cooper M. A., Hoy W. E. (2011). Biomarkers in chronic kidney disease: a review. *Kidney International*.

[94] Martinez B., Peplow P. V. (2020). MicroRNAs in blood and cerebrospinal fluid as diagnostic biomarkers of multiple sclerosis and to monitor disease progression. *Neural Regeneration Research*.

[95] Tayel S. I., Saleh A. A., El-Hefnawy S. M., Elzorkany K. M. A., Elgarawany G. E., Noreldin R. I. (2020). Simultaneous Assessment of MicroRNAs 126 and 192 in Diabetic Nephropathy Patients and the Relation of these MicroRNAs with Urinary Albumin. *Current Molecular Medicine*.

[96] Kim H., Bae Y. U., Jeon J. S. (2019). The circulating exosomal microRNAs related to albuminuria in patients with diabetic nephropathy. *Journal of Translational Medicine*.

[97] Prabu P., Rome S., Sathishkumar C. (2019). MicroRNAs from urinary extracellular vesicles are non-invasive early biomarkers of diabetic nephropathy in type 2 diabetes patients with the 'Asian Indian phenotype'. *Diabetes & Metabolism*.

[98] Conserva F., Barozzino M., Pesce F. (2019). Urinary miRNA-27b-3p and miRNA-1228-3p correlate with the progression of kidney fibrosis in diabetic nephropathy. *Scientific Reports*.

[99] Meng L., Li G., Liu X., Jiang J., Zhu M., Sun Y. (2018). Decreased urine miR-199-3p may be a potential biomarker for diabetic nephropathy via targeting zinc finger E-box-binding protein 1. *Clinical Laboratory*.

[100] Beltrami C., Simpson K., Jesky M. (2018). Association of elevated urinary miR-126, miR-155, and miR-29b with diabetic kidney disease. *The American Journal of Pathology*.

[101] Yu S., Zhao H., Yang W. (2019). The alcohol extract of *Coreopsis tinctoria* Nutt ameliorates diabetes and diabetic nephropathy in db/db mice through miR-192/miR-200b and PTEN/AKT and ZEB2/ECM pathways. *BioMed Research International*.

[102] Sun Z., Ma Y., Chen F., Wang S., Chen B., Shi J. (2018). miR-133b and miR-199b knockdown attenuate TGF-*β*1-induced epithelial to mesenchymal transition and renal fibrosis by targeting SIRT1 in diabetic nephropathy. *European Journal of Pharmacology*.

[103] Civantos E., Bosch E., Ramírez Bustillo E. (2017). Sitagliptin ameliorates oxidative stress in experimental diabetic nephropathy by diminishing the miR-200a/Keap-1/Nrf2 antioxidant pathway. *Diabetes, Metabolic Syndrome and Obesity: Targets and Therapy*.

[104] Xue M., Cheng Y., Han F. (2018). Triptolide attenuates renal tubular epithelial-mesenchymal transition via the MiR-188-5p-mediated PI3K/AKT pathway in diabetic kidney disease. *International Journal of Biological Sciences*.

[105] Wu L., Wang Q., Guo F. (2016). MicroRNA-27a induces mesangial cell injury by targeting of PPAR*γ* and its in vivo knockdown prevents progression of diabetic nephropathy. *Scientific Reports*.

[106] Wu D., Cheng Y. G., Huang X., Zhong M. W., Liu S. Z., Hu S. Y. (2018). Downregulation of lncRNA MALAT1 contributes to renal functional improvement after duodenal-jejunal bypass in a diabetic rat model. *Journal of Physiology and Biochemistry*.

[107] Nagaishi K., Mizue Y., Chikenji T. (2016). Mesenchymal stem cell therapy ameliorates diabetic nephropathy via the paracrine effect of renal trophic factors including exosomes. *Scientific Reports*.

